# Publisher’s Note: A New Chapter for the *Journal of Market Access and Health Policy* (*JMAHP*)—Continued Publication by MDPI

**DOI:** 10.3390/jmahp12010001

**Published:** 2024-01-01

**Authors:** Clàudia Aunós

**Affiliations:** MDPI AG, St. Alban-Anlage 66, 4052 Basel, Switzerland; claudia.aunos@mdpi.com

The *Journal of Market Access and Health Policy* (*JMAHP*) is an open access peer-reviewed publication focused on Market Access and its sub-dimensions. It is owned by and the official journal of the Market Access Society (MAS) [1].

*JMAHP* was released in August 2013 as a goal to provide an extended forum on the broad concept of “market access”, including technical, sociological, economic, scientific, psychological, and policy perspectives [2].

Prof. Dr. Mondher Toumi has been Editor-in-Chief of *JMAHP* since its inception. He has created a successful platform to share knowledge and insights for researchers, policymakers, and industry professionals alike. Through the efforts of Prof. Dr. Toumi and his editorial team, the journal is now indexed in key databases like Scopus and PubMed [3,4]. 

From 2017 to 2023, the journal had been published in Taylor and Francis as an annual journal, and as of Volume 12, 2024, the *Journal of Market Access and Health Policy* will be published by MDPI [5] as a quarterly journal. As a courtesy and upon agreement with Taylor & Francis, previous articles of the journal will also be hosted by MDPI on the journal’s website.

It will continue to be an evolving and innovative journal, serving the scientific community and industry professionals by publishing high-quality, open access, and peer-reviewed articles on all aspects of Market Access.

We would like to offer a warm welcome to all Editorial Board Members, the Market Access Society members, and authors. We hope that the *Journal of Market Access and Health Policy* will continue to serve as a platform for sharing advances in the field of Market Access and health policies.
